# Supplementary material for: Acceptance and compliance with micronutrient powder (MNP) among children aged 6–23 months in northern Nigeria
Source: PLOS Glob Public Health. 2022 Oct 17;2(10):e0000961. doi: 10.1371/journal.pgph.0000961 (PMC10022258; doi:10.1371/journal.pgph.0000961)
Supplement: S1 Dataset — (ZIP) [file pgph.0000961.s008.zip › S8_Dataset/Direct Observations.zip!/Direct Observations/120715_K_DW_AD_2_DO.pdf]

1.1.1 Instrument F –Direct Observation Form

**<<Instrument F Direct Observation Form>>**
**PI Name:** PragyaMathema,Stephen Kodish<br>**Study Title:** Formative research to develop an infant and young child feeding (IYCF) intervention with micronutrient powder (MNP) for children aged 6 – 23 months<br>**PI Version/Date:** Version1.0/January 24, 2015

Date: 12/07/15 Location: Rafin gora

Data Collector: Amina Duniya

Main goals of this direct observation are to answer the following questions:<br><br>1. In this household, what would be the best way(s) to give nutritional supplements to young children? Why?<br><br>2. In this household, describe the food sharing that you observe throughout the meal. Are there any predictable patterns?<br><br>3. In this household, who is favored and how is this favoritism expressed during meal preparation and eating?

Household Composition Table (Fill out as you do data collection)

INITIALS	SEX (M/F)	AGE (estimated)	Relationship to household	Description (not necessary)
BM	F	25years	Mother of the baby	Slim, dark in complexion with tribal marks, wearing a blue and cream blouse, tying a blue wrapper
BG	F	17months	Baby girl of the house	Dark in complexion, wearing a blue top and green short with blue earrings
SB	M	4years	Senior brother to BG	Dark in complexion and wearing a blue kaftan
BL	M	20years	Brother in-law	Very dark in complexion, wearing a red top and black trouser
NW	F	27years	neighbor	Dark in complexion, pregnant and tying a blue wrapper on her chest
FH	M	40years	Father of the household	Tall not so dark in complexion, wearing a purple kaftan top and cream trouser

PARTICIPANT ID: _________

TIME	ACTOR INITIALS	RECIPIENT INITIALS	WHAT HAPPENED	FOOD	Reactivity
10:00	BM	BG	Strapped BG to her back, BG is sick		
10:05	BM	BG	Still strapped to mother's back		
10:10	BG		Sleeping		
	BM		Washing clothes		
10:20	BM		Still washing while BG Is still strapped to her back		
10:30	BG		Wakes up and begins to cry		
	BM	BG	Brings her down and keeps her on the mat		
10:35	BG		Lies down on the mat		
10:40	BM	BG	Gives her tuwo and okra soup	tuwo and okra soup	
10:45	BG		Still eating	tuwo and okra soup	
	BM	BG	Still eating	tuwo and okra soup	
10:50	SB		Starts to cry for food too		
	BM	SB	Tells him to wait for BG to finish eating		
	BG		BG finishes eating	tuwo and okra soup	
10:55	BM	SB	Gives the remaining food to SB to eat		
	BG		sleeping		
11:00	SB		Still eating		
	BG		Sleeping		

TIME	ACTOR INITIALS	RECIPIENT INITIALS	WHAT HAPPENED	FOOD	Reactivity
	SB		Finish eating and BM carries the plate to her room		
11:05	BG		Still sleeping		
	BM		Watching BG as she sleeps		
11:10	BB		Still sleeping		
	BB		Wakes up and starts crying		
	BM		Gives BG water to drink		
11:15	BB		Goes back to sleep		
11:20	BG	BM	Wakes up, starts crying and goes to lie down on mother's lap		
	BG		Continues sleeping on BM's lap		
11:25	BG		Still sleeping		
11:30	BG		Still sleeping		
11:40	BG		Still sleeping		
	BM		Watching BG while she sleeps		
11:50	BG		Still sleeping		
	BM		Lies down next to baby girl but not sleeping		
12:00	BG		Still sleeping		
12:15	MF		Comes into the house and BM gets up		
	BM	MF	Gisting while BG sleeps		
	BG		Still sleeping		
	BM		Still gisting		

TIME	ACTOR INITIALS	RECIPIENT INITIALS	WHAT HAPPENED	FOOD	Reactivity
12:25	BG		Still sleeping		
12:30	BG		Still sleeping		
	BM	MF	Still gisting		
12:40	BG		Still sleeping		
	BM	MF	Still gisting		
	BG		Wakes up and begins to cry		
	BM	BG	Carries her on her back and rocks her to sleep		
12:45	BG		Goes back to sleep on BM's lap		
	BM	MF	Still gisting with MF		
12:50	BG		Still sleeping		
12:55	BG		Still sleeping		
	BM	BG	Puts BG back on the mat to sleep		
	BM	MF	Continues gisting with MF		
1:00	BG		Still sleeping		
1:05	BG		Still sleeping		
	MF		Gets up to leave		
	BM	MF	Sees her close to door, comes back and sits close to BG		
1:10	BG		Still sleeping		
1:20	BG		Still sleeping		
	BM	BG	Using a piece of plastic fan to blow baby because she was sweating		

TIME	ACTOR INITIALS	RECIPIENT INITIALS	WHAT HAPPENED	FOOD	Reactivity
	SB		Comes back and lies down on the mat to sleep		
1:25	SB	BM	Askes BM for water and BM agrees		
	BG		Also wakes up from sleep		
	BM	BG	Gives her water to drink		
1:30	BM	BG	Gives he cold tuwo and okra to eat without washing the soup bowl	Tuwo and okra soup	
	BG	BM	Tells mother she needs to use the toilet		
	BM	BG	Takes he rto front to the house to defecate on the floor		
	BM		Leaves the food on the floor with flies perching on it		
1:35	BM	BG	BG finishes defecating and BM her up with water, brings her into the room and places her on the mat		
	BM		Goes back to pack the feaces and throws it into the pit toilet		
	BG		Defecates again on the mat		
	BM	BG	Picks her from the mat to the floor to continue		
	BM		Carries the mat and keeps outside		
	BG		Defecates a little on the floor in the room		
	BM	BG	Takes her outside to continue		
1:40	BM		Pours sand on the faeces in the room, and used a broom to pack it		
	BM		Goes to wash BG's buttocks after she was done		
	BM	BG	Brings her inside and drops her on another mat		
	BM		Goes back outside to pack the faeces BG dropped outside		
	BM	BG		tuwo and	

TIME	ACTOR INITIALS	RECIPIENT INITIALS	WHAT HAPPENED	FOOD	Reactivity
1:45			Comes back abd begins to feed BG with tuwo and okra soup without hand washing	okra soup	
	BG	BM	Finish feeding BG and gives her some water to drink		
	BM		Pours some of the water in a dirty cup and washes her hand with it		
1:50	BG		Asks for more food		
	BM	BG	Carries the food again and gives BG to eat by herself		
	BB		Begins to eat the food on BM's lap with dirty hands		
	BG		Asks for water to drink		
	BM	BG	Gives her water to drink		
	BG		Continues eating and ask for water		
	BM	BG	Gives her some more water to drink		
1:55	BG		Finished eating and ask for water		
	BM	BG	Gives her more water to drink		
	BM	BG	Pours the remaining water into the empty soup bowl and washes BG's hands with it		
	BM	BG	Drops baby on the mat and washes the soup bowl with the water used in washing BG's hand		
2:00	BM		Throws the water outside the door		
	BM		Also lies down on the mat close to BG and SB		
2:05	BG		Lying down on the mat not sleeping but SB is sleeping		
	BM		Using her head tie to drive away flies from BG and SB		
	BG		Still lying down but not sleeping		

TIME	ACTOR INITIALS	RECIPIENT INITIALS	WHAT HAPPENED	FOOD	Reactivity
2:10					
	BG		Starts sleeping		
2:20	BG		Still sleeping with SB		
	BG		Wakes up and asks for water		
	BM	BG	Gives her water to drink		
2:30	BG		Goes back to sleep		
2:40	BB		Still sleeping		
	BM		sleeping		
2:45	BG		Wakes up and asks for water		
	BM	BG	Gives her water to drink		
2:50	SB		Wakes up and starts to cry		
	BM		Goes out of the room		
	SB		Stops crying but still lying down (looking hungry)		
	BG		Starts crying		
	BM		Continues what she is doing outside		
2:55	BM		Comes in with a left over tuwo, which she mixed with water and added sugar		
	B M	BG	Feeds BG with it while SB starts to cry, tells him to get a spoon		
	SB		Goes and comes back with a spoon and also begins to feed from the gruel while BM feeds BG		
3:00	BM	BG	Still feeding BG while SB still drinks from the gruel		
3:05	BG		Stops drinking		
	SB		Continues to drink		

TIME	ACTOR INITIALS	RECIPIENT INITIALS	WHAT HAPPENED	FOOD	Reactivity
3:10	SB		Finish drinking and lies down on the mat		
	BG		Comes down from BM's lap and goes outside to defecate again on the floor		
3:15	BM	BG	BG finishes and BM washes her up and brings her to the room		
	BM		Packs the feaces from the floor and throws into the toilet		
3:20	BM	BG	Carries BG and straps her to her back with a wrapper		
	BM		Drinks the remaining gruel left	Gruel made from tuwo	
3:25	BM		Finished drinking the gruel		
	BG		Begins to fuss on mother's back		
	BM	BG	Comforts her while she keeps quiet		
3:30	BM	BG	Brings her down and carries her on her laps		
3:35	BM	BG	Still carrying her on her laps		
3:40	BG		Comes down from BM's laps and begins to play		
	BG		Starts crying and climbs back to mother's lap		
3:45	BG		Still on mother's lap		
	BL		Comes back from the farm and sits with BM		
	NW		Comes into the house and sits with BM and BL		
3:50	BM		Gisting with BL and NW		
	BG		Still lying down on mother's lap		
3:55	SB		Wakes up and starts crying		

TIME	ACTOR INITIALS	RECIPIENT INITIALS	WHAT HAPPENED	FOOD	Reactivity
	BM	SB	BM tells him to stop crying and he does		
	BM		Still lying on BM's laps		
4:00	BM	BG	Goes into the room and brings 'HEKIDORO' vegetable, mixed with palm kernel oil and salt an dgives BG to eat	Hekidoro vegetable	
	SB		Still crying and BM does not ans him	Hekidoro vegetable	
4:10	BG		Still eating 'hekidoro'		
	SB		Still crying and running a temperature		
	BM		Gives BG tuwo dawa to eat with hekidoro	Tuwo dawa and hekidoro	
4:15	BM	SB	Tells him to eat some food but he refuses		
	BM	SB	Gives him paracetamol to drink		
4:20	BG		Still eating	Tuwo dawa and hekidoro	
	SB		Still crying		
4:25	BG		Still eating	Tuwo dawa and hekidoro	
	BM	BG&SB	Gives them some biscuit to eat	biscuit	
4:30	SB		Finish eating biscuit and lies down on the mat		
	BG		Still eating biscuit	Biscuit	
4:35	BG		Finish eating biscuit and begins to play		
4:40	BM		Comes into the house with 'kuka powder' and begins to sieve it		
	BG	BM	Starts crying for BM to carry her		

TIME	ACTOR INITIALS	RECIPIENT INITIALS	WHAT HAPPENED	FOOD	Reactivity
	BM	BG	Straps BG to her back		
4:45	BM		Still sieving ‘kuka’ leaves with BG strapped to her back		
4:50	FH		Comes back from the farm		
	BM		Goes to fetch water from the well with BG still strapped to her back		
	BM		Keeps water in the bathroom for FH to take his bath and continues sieving		
	BM	BG	Still strapped to her back		
4:55	BM		Still sieving		
			Finish sieving and goes out to pound the pellets of kuka leaves still remaining. BG still strapped to her back		
5:00	BM		Finish pounding and continues to sieve kuka with BG strapped to her back		
5:05	BM		Goes out to fetch water and firewood with BG strapped to her back		
	BM		Comes back and goes out to the bore hole in the community to fetch water while baby is strapped to her back		
5:10	BM		Waiting for her turn at the borehole, BG still strapped to her back		
5:15	BM		Still Waiting for her turn at the borehole, finally gets to fetch water		
5:20	BM		Walks back home with bucket of water on her head while baby is still strapped to her back		
	BM	BG	Drops her on the mat and goes to wash some clothes		
5:25	BM		Still washing		

TIME	ACTOR INITIALS	RECIPIENT INITIALS	WHAT HAPPENED	FOOD	Reactivity
	BG		Lying down on the mat and sleeping		
5:30	BM		Still washing clothes		
	BG		Still sleeping		
5:35	BM		Still washing clothes		
	BG		Still sleeping		
5:45	BM		Finish washing the cloth and puts them on a line to dry		
	FH		Comes in with some drugs for BG and gives them to BM.<br>FH bought the drugs from the chemist, he was asked		
5:50	BM	BG	Wakes baby fro sleep and gives her the drugs to take		
	BM	BG	Gives her a capsule of quinne syrup and a capful of paracetamol		
5:55	BM	FH	Gives BG to her father to hold while she goes to keep the drugs inside the bedroom		
	BM		Comes back with some water to give BG to drink		
6:00	BM		Collects ger from FH and straps her to her back and goes back to prepare the meal		
6:05	BM		Puts water on the fire with pepper, locust bean, potash and salt to boil		
6:15	BM		Adds ‘kuka’ leaves and mixes it together		
6:20	BM		Allows it to boil for 5minutes and brings it down		
6:25	BM		Puts another pot of water to boil to makes tuwo dawa		
6:30	BM		Adds flour paste to boiling water and mix thoroughly		
6:35	BM		Allows to cook for 5 minutes and brings it down		
6:40	BM		Dishes food into 4 different plates	Tuwo	

TIME	ACTOR INITIALS	RECIPIENT INITIALS	WHAT HAPPENED	FOOD	Reactivity
				and miyan kuka	
	BM		Carries the plate to the sitting room and calls FH,SB and BG to eat		
6:50	BM	FH	Served him his food seperately		
	BM	SB	Tells him to pick his food from the tray		
	BM	BG	Carries her food and that of BG and sits at one corner of the house where they both eat with BM monitoring BG to eat		

NOTE

BG has been sick for a week and husband orders she should not be given MNP until she gets better.

Primary Questions (Complete after you complete data collection):

From what you have seen observing THIS HOUSEHOLD, please answer the following questions in detail:

Question	Write your detailed response here
1. In this household, what would be the best way(s) to give nutritional supplements such as an MNP to young children to ensure compliance?<br><br>Why?	The best way is through the mother because eshe is always with the child and monitor’s her eating
2. In this household, who is favored during meal preparation or feeding?	The baby is favored more because during the meal time, the mother paid several Attention to her

How is this favoritism expressed?	
3. How could we promote the MNP supplement for this family that would attract their attention?<br><br>Who would they listen to?	By educating them on the benefit of the MNP to the child
4. In this household, what would be the best ways to promote the MNP but limit how much it would be shared?<br><br>Why?	By advising mothers on the need to give only the child the nutrient is meant for, if not the child will not get any nutrient
5. What other issues and	There is a problem of hygiene, there was no proper hand washing

concerns did you see with this family that are important for us to consider with regard to introduction of an MNP?<br><br>Why?	practices prior to eating
6. Based on your experience observing this child today, do you believe reactivity negatively impacted your ability to see the food-related behaviors of the child?<br><br>Please explain your answer in detail.	No I don't think so
